# Determination of Tumor Marker Carcinoembryonic Antigen with Biosensor Based on Optical Quantum Weak Measurements

**DOI:** 10.3390/s18051550

**Published:** 2018-05-14

**Authors:** Tian Guan, Xiangnan Wang, Dongmei Li, Yilong Zhang, Yonghong He, Lixuan Shi, Yiqing Liu, Yuxuan Yang, Yang Xu, Rui Cui

**Affiliations:** 1 Department of Biomedical Engineering, School of Medicine, Tsinghua University, Beijing 100084, China; wxn16@mails.tsinghua.edu.cn (X.W.); liu-yq17@mails.tsinghua.edu.cn (Y.L.); yx-yang16@mails.tsinghua.edu.cn (Y.Y.); 2 Shenzhen Key Laboratory for Minimal Invasive Medical Technologies, Institute of Optical Imaging and Sensing, Graduate School at Shenzhen, Tsinghua University, Shenzhen 518055, China; ldm13@mails.tsinghua.edu.cn (D.L.); zhangyilong@zjut.edu.cn (Y.Z.); slx16@mails.tsinghua.edu.cn (L.S.); xuyang17@mails.tsinghua.edu.cn (Y.X.); 3 Department of Physics, Tsinghua University, Beijing 100084, China; 4 Shenzhen Maternity & Child Healthcare Hospital, Shenzhen 518055, China; yiwuke@126.com

**Keywords:** phase measurement, optical biosensor, weak measurement, total internal reflection

## Abstract

A phase-sensitive weak measurement biosensor was proposed for the detection of carcinoembryonic antigen (CEA), one common category of tumor markers. The total internal reflection (TIR) at the interface of the prism without precious metal coating was exploited to introduce the phase delay between horizontal and vertical polarizations, which can be determined through the central wavelength shift of output spectra for the sensing of the refractive index of the sample. In the weak measurement analysis, the specific binding reaction of tumor markers with a refractive index change on the surface of the prism can be monitored in real time through the central wavelength shift. With the specific absorption measurement, the feasibility of this weak measurement-based biosensor was experimentally demonstrated. We provide a low cost and convenient approach for tumor marker detection.

## 1. Introduction

Tumor markers are always found in blood, urine, or body tissues, and they present a higher level in patients with one or more types of cancer compared with normal patients [1]. The ultra-sensitive detection of tumor markers contributes to the early diagnosis and clinical treatment of tumors [2]. In recent years, greater attention has been paid to the detection of tumor markers for the purpose of early diagnosis and treatment, which is of profound significance to cancer patients. For this reason, technology that can operate in real time at high precision for the detection of tumor markers would be useful.

Current methods for the detection of tumor markers, including electrochemistry [3,4,5], fluorescence [6,7,8], chemiluminescence [9,10], and graphene-based biosensors [11,12,13], provide sensitive and specific techniques. However, some of them need expensive reagents, bulky instruments, and lack the traits of real-time and label-free detection. Moreover, the technical problems, including multiple reaction steps, high time consumption, and the certain possibility of false results due to nonspecific adsorption, need to be solved.

In recent decades, phase-sensitive optical biosensors with reflective configurations have become an intensely researched subject due to their simple structures and ability to perform label-free detection in real-time. For instance, surface plasmon resonance (SPR)-based biosensors can realize direct monitoring of molecular interactions with the trait of label-free, SPR biosensors have been extensively used to monitor molecular interactions because of their outstanding sensitivity, reliability, and reproducibility [14,15,16]. Li et al. developed an SPR biosensor that allows the label-free detection of CEA, during which accurate gold film coatings thickness control and optimization of the incident angle and wavelength is required [17].

In recent years, weak measurement has attracted increasing attention as a weak signal enhancing metrology. It has a variety of applications in precision detection, such as sub-pulse width temporal delay [18], polarization rotation [19], light beam deflection [20], temperature [21], and the Goos–Hänchen shift [22]. The weak measurement in frequency domain is a technique based on optical phase modulation which is caused by the sample in the system and demodulation with output spectra. Weak measurement has been used in biosensing and has showed significant improvements in several applications, such as the assessment of high precision in glucose concentration [23], real-time MIP (molecularly imprinted polymer) sensor [24], and sensitive chiral sensor with enantioselectivity [25]. In biomolecule detection, the measuring signal is always weak. Hence, the design of TIR (total internal reflection)-based weak measurement system with common path configuration is applicable for label-free biomolecule sensing.

In this work, we introduce the method of weak measurement to the detection of tumor markers for the first time. We present a simple TIR-based weak measurement biosensor with common path configuration for label-free tumor marker detection. The interaction of the tumor markers on the surface of the silica prism induces changes in the refractive index of the sample. Based on Fresnel’s formula, the total internal reflection (TIR) at the interface of the prism is exploited to introduce the phase difference of the eigenstates of the weak measurement system, which was realized by the two orthogonal polarizations. The central wavelength shifts of output spectra, which are collected in real time with the procedure on the computer, is exploited for the quantitative analysis of the phase difference between horizontal polarization and vertical polarizations, corresponding to the refractive index of the sample solution. The capture process of the tumor markers, in which the recognition sites of the anti-CEA catch the target CEA, can induce changes in the refractive index of the reflected surface. Consequently, the phase difference between the horizontal and vertical polarizations varies with the refractive index change of the sample. Therefore, the capture process can be monitored in real time through the central wavelength shifts of output spectra. Unlike some conventional methods of detecting tumor markers, the label-free detection based on weak measurement can be performed directly on the surface of the glass without precious metal coating. The transparent prism without a surface coating makes this method easy to use with microscopy, and it can be expanded for multichannel detection. It is experimentally demonstrated that the weak measurement biosensor exhibits specificity, sensitivity, and label-free and real-time detection for CEA. Other tumor markers can also be detected with this protocol, which exhibits a great potential and significance to the screening, diagnosis, and treatment of cancer.

## 2. Materials and Methods

### 2.1. Subsection

Phosphate-buffered saline (PBS) were bought from Sigma Aldrich, Inc. (St. Louis, MO, USA). Human CEA and monoclonal human anti-CEA were acquired from XMa Life Science, Inc. (Saco, ME, USA). Prostate-specific antigen (PSA), carbohydrate antigen 199 (CA199) and carbohydrate antigen 125 (CA125) were purchased from XMa Life Science, Inc. (Saco, ME, USA). 95% H_2_SO_4_ and 30% hydrogen peroxide H_2_O_2_ were purchased from TianJin BaiShi Chemical Co., Ltd. (Tianjin, China). Refractive index matching liquid, 90% HCl, Tris-HCl, and dopamine hydrochloride (98%) was purchased from Aladdin (Shanghai, China). Milli-Q water (18.2 MΩ·cm, Millipore Corp., Bedford, MA, USA) was used in the experiments. All other organic solvents and chemical reagents were purchased from ShenzhenTianxiang Huabo Co., Ltd. (Shenzhen, China). SF6 glass slide and prism were purchased from FuZhou Aerfa optics (Fuzhou, China).

#### 2.1.1. Cleaning of Substrate and Prism

The SF6 glass substrate and prism were cleaned with a Piranha solution mixed solvent of H_2_SO_4_ and H_2_O_2_ at a ratio of 3:7. Subsequently, the SF6 glass substrate and prism were rinsed with deionized water. Next, the SF6 glass substrate and prism were dried with nitrogen purging.

#### 2.1.2. Preparation of the Dopamine Solution

An amount of 0.121 g (1 mmol) of Tris-HCl was dissolved in 100 mL of deionized water. Subsequently, the solution was titrated to pH 8.5 with diluted 10% HCl solution. An amount of 0.04 g of dopamine powder was dissolved in 20 mL of 8.5 pH Tris buffer to obtain a 2 mg/mL dopamine-Tris solution. Because dopamine is oxidized at 8.5 pH and self-polymerization occurs immediately, the dopamine solution needs to be kept in shade.

#### 2.1.3. Preparation of the Tumor Markers

Preparation of CEA and anti-CEA solution: 1 mg of CEA was dissolved in 10 mL of 10 mM 7.4 pH PBS buffer. Subsequently, 100 μg/mL CEA is diluted to different concentrations with 10 mM of PBS at pH 7.4. The same method was utilized to produce anti-CEA solution, PSA solution, Ca125 solution, and Ca199 solution.

### 2.2. Experimental Setups

**Figure 1:**
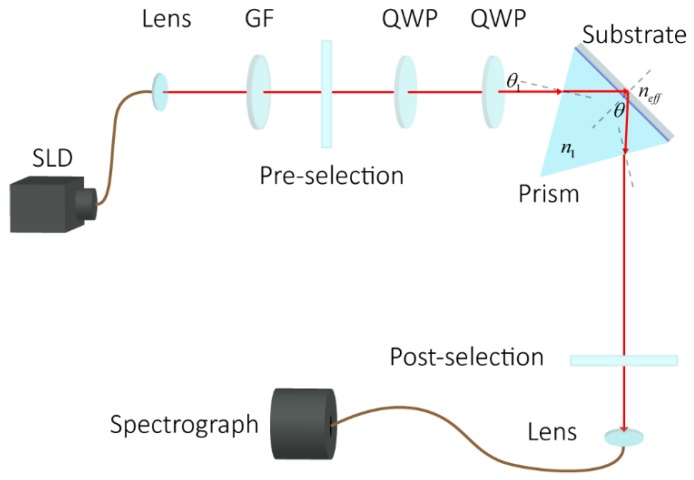
The schematic diagram of the weak measurement system. SLD: super luminescent diode central at 830 nm with a bandwidth of 50 nm. GF: Gaussian filter with a bandwidth of 20 nm. QWP: quarter wave plate.

In this work, the schematic diagram of the TIR based weak measurement system is shown in Figure 1. The incident light from SLD (IPSDS0803, 5 mW, Inphenix, Wuhan, China) centered at 830 nm, and propagated through a Gaussian filter with a bandwidth of 10 nm. It was then preselected by the first linear polarizer (Thorlabs Inc., LPVIS050-MP2, Shanghai, China, an extinction ratio of 100,000:1) with an angle of α, which was the angle between the polarization axis of the first polarizer and the horizontal direction. The light beam then passed through a pair of quarter-wave plates with fast axes perpendicular to each other, which was used for continuous phase adjustment. The SF6 prism was placed under the SF6 substrate with refractive index matching liquid. The SF6 substrate was utilized for easily replacement. The light was then reflected by the SF6 substrate. The reflected light was post-selected by the second linear polarizer. Finally, the output spectra signal was collected by a spectrograph (OceanOptiocs, HR2000, Shanghai, China).

The interaction of the samples on the surface of the substrate induced the refractive index changes of the liquid samples. The total internal reflection (TIR) at the interface of the substrate was exploited to introduce the phase difference of the eigenstates of the weak measurement system, which was actualized by two orthogonal polarizations. The central wavelength shift of output spectra, which was collected in real time with the spectrograph connected with the computer, was exploited for the quantitative analysis of the phase difference between the two polarizations, corresponding to the refractive index of the liquid sample. Therefore, the reaction process in the liquid sample can be monitored in real time through the central wavelength shift of the output spectra.

This system works with common path implementation. Compared with Mach-Zehnder and other non-common path system, it shows a stronger anti-jamming capability. Because the surface of the prism serves as the area of detection, it is convenient to employ real-time and label-free tumor marker detection. In addition, the TIR-based weak measurement biosensor, unlike SPR sensors, makes it possible to monitor the immunoreaction on the surface of the reflecting prism directly without accurate gold film coatings.

### 2.3. Weak Measurement Theory

The weak measurement system is shown in Figure 1. The states of the system could be definitely expressed. According to the angle of the preselected polarizer, the preselection state is given by $|\psi_{i}\rangle =\sin\alpha |H\rangle +\cos\alpha |V\rangle$. The light passes through two quarter-wave plates whose fast axes are perpendicular to each other. When one of the two quarter-wave plates is tilted, the phase difference between the H and V polarization changes. The two quarter-wave plates are used to continuously increase the phase difference between H and V polarizations by tilting the first wave plate at small angle χ. The relationship between the phase difference δ and the inclination angle χ can be obtained as

(1) $$\delta =\pm \frac{\pi \left(n_{e}-n_{o}\right)h\chi^{2}}{\lambda }.$$

In Equation (1), $n_{e}$ and $n_{o}$ denotes the refractive index for the extraordinary and ordinary components, *h* is the thickness of the wave plate, and λ is the wavelength of the light.

In this work, the refractive index *n* of the prism was 1.75 RIU. Hence, with the expression $\sin^{-1}\left(1/n\right)$, the total internal reflection angle of the prism can be calculated to be 49.46°. In the experiment setup, the light was reflected by the prism with an incident angle of 50°. When the solution flowed through the surface of the substrate surface, the effective refractive index of the substrate surface changed. Thus, the phase difference between the H and V polarization components of the reflected light changed. According to Fresnel’s Formula, the phase difference caused by the refractive index change is expressed as

(2) $$\Delta =2\tan^{-1}\frac{\sqrt{{\left(n_{eff}/n_{1}\right)}^{2}\sin^{2}\theta_{1}-1}}{n_{eff}/n_{1}\sin\theta_{1}\tan\theta_{1}}.$$

$n_{eff}$ is the effective refractive index of the substrate surface, $n_{1}$ is the refractive index of the prism, and $\theta_{1}$ is the incident angle. Hence, the total phase difference between H and V polarization could be expressed as $\Delta +\delta =\Delta +a\chi^{2}$.

The light passes through the second polarizer which is used to prepare the post-selection state with its polarization axis placed at angle of π/2+β with the first polarizer’s axis. The post-selection state is given by $|\psi_{f}\rangle =\cos\left(\alpha +\beta \right)|H\rangle +\sin\left(\alpha +\beta \right)e^{i\left(\Delta +\delta \right)}|V\rangle$.

The observable operator was A=|H〉〈H|−|V〉〈V|. The weak value $A_{w}$ can be expressed as

(3) $$A_{w}=\frac{\langle \psi_{f}\left|\hat{A}\right|\psi_{i}\rangle }{\langle \psi_{f}|\psi_{i}\rangle }=\frac{\sin\alpha \cos\left(\alpha +\beta \right)+\cos\alpha \sin\left(\alpha +\beta \right)e^{i\left(\delta +\Delta \right)}}{\sin\alpha \cos\left(\alpha +\beta \right)-\cos\alpha \sin\left(\alpha +\beta \right)e^{i\left(\delta +\Delta \right)}}.$$

We define γ=cotαtan(α+β), so the imaginary part of weak value could be simplified as

(4) ImAw=1+γei(δ+Δ)1−γei(δ+Δ)≈2γsin(δ+Δ)1+γ2−2γcos(δ+Δ).

Depending on the relationship between the momentum and the imaginary part of the weak value $\delta P=2k{\left(\Delta P\right)}^{2}$ [26], we use P=2π/λ to achieve the quantitative expression of central wavelength shift with respect to phase change:

(5) δλ=−2πk(Δλ)2λ0ImAw=−4πk(Δλ)2γsin(δ+Δ)λ0(1+γ2−2γcos(δ+Δ)).

According to $\Delta +\delta =\Delta +a\chi^{2}$, we can obtain the relationship between the shift of the center wavelength and the dip angle of the quarter-wave plate:

(6) δλ=−2πk(Δλ)2λ0ImAw=−4πk(Δλ)2γsin(aχ2+Δ)λ0(1+γ2−2γcos(aχ2+Δ)).

**Figure 2:**
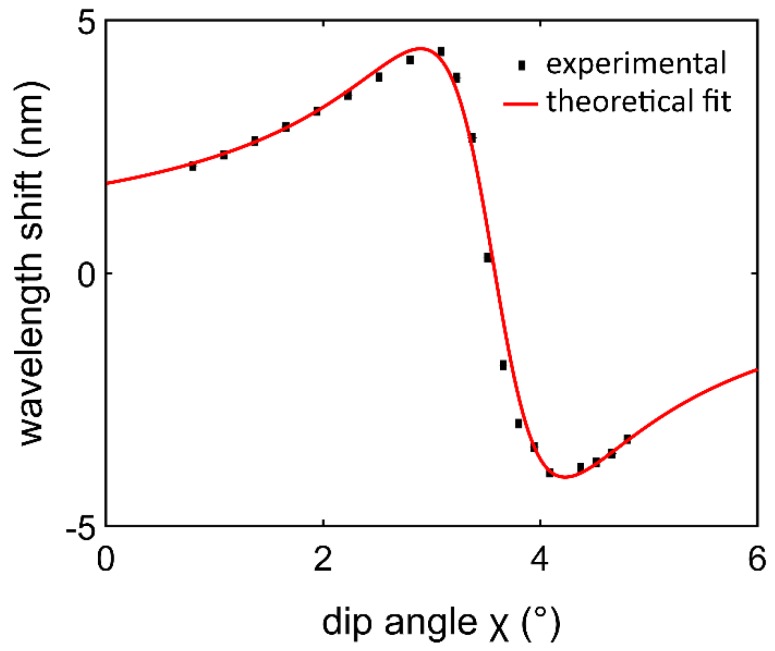
The central wavelength shifts with respect to the dip angle *χ*. The solid line is the theoretical expectation. The square dots are the experimental data; the standard deviation of experimental data is too small to be distinguished.

To demonstrate the principle of weak measurement in this scheme, the theoretical and experimental response curve of the central wavelength shift with the dip angle change of the quarter-wave plate is acquired. MATLAB (MathWorks, Inc., Natick, MA, USA) is utilized to fit Equation (6) to implement the theoretical simulations. The dip angle of the quarter-wave plate was presented as the *x*-axis, and the *y*-axis shows the central wavelength shift. Central wavelength $\lambda_{0}$ is 830 nm, Δλ is 10 nm, α=45°, d and β=0.45°. The theoretical result is shown in Figure 2 (solid line). The relationship between the shift of the central wavelength (*δλ*) and the angle χ changed from 0 to 6° is shown in Figure 2 (square dots).

The result is shown in Figure 2. When the dip angle increased, the phase difference between H polarization and V polarization changed. δλ changed slowly until it reached a maximum, and then δλ decreased sharply to a minimum. For this rapidly changing region, the response is sharp and relatively linear. Hence, in this internal of dip angle change, the central wavelength shift is sensitive to the angle corresponding to the phase difference. Thus, the phase difference can be detected with this linear range as the working point.

## 3. Results and Discussion

**Figure 3:**
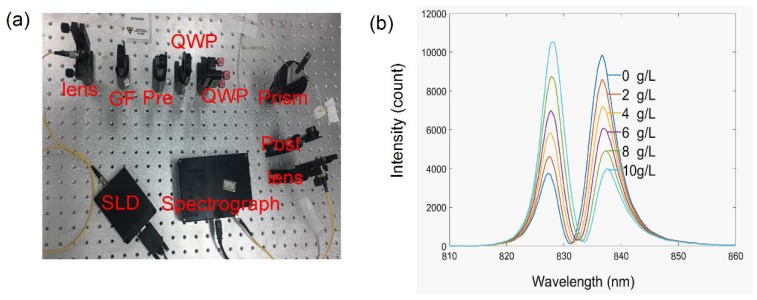
(**a**) The photograph of the real system. SLD: super luminescent diode central at 840 nm; GF: Gaussian filter with a bandwidth of 20 nm; Pre: pre-selection polarizer; QWP: quarter wave plate; post: post-selection polarizer. (**b**) The experimentally detected two-peak spectrum.

The photograph of the real system is shown as Figure 3a. Superluminescent diode centered at 830 nm was used to provide the wide band light source. The incident light was shaped by a Gaussian filter (GF) and then preselected by the first linear polarizer. The quarter wave plates are used to adjust the phase difference between horizontal and vertical polarized components for the high precision measurement. Subsequently the light is reflected by the prism. The post-selection of this weak measurement system is produced by the second linear polarizer. The output light beam is received by the spectrograph, which is connected with a computer for spectral analysis.

A series of diluted glucose solution experiments were carried out to demonstrate the working principle of the system. First, the wave plate is tilted to set the work point in the sharp area shown in Figure 2. Subsequently, a series of diluted glucose solutions with concentrations of 0–10 g/L are injected into the flow channel on the surface of the prism, causing a change from the initial phase. After that, the spectra captured by the spectrograph are smoothed by an FFT filter to reduce the high-frequency noise corresponding to the glitch of light source and shot noise. The integration time of the spectrograph is 5 ms. A MATLAB program is established to acquire a real-time signal of spectrum shifts corresponding to the concentrations of the glucose solutions. The shifts are obtained by calculating the expectation of the spectra. At the beginning, the spectrum is Gaussian shaped, centered at 830 nm. Because the interactions cause a longitudinal shift, the spectrum differs from the original Gaussian shape. As predicted theoretically [27], the experimentally detected two-peak spectra are shown in Figure 3b. Phase difference was caused by the refractive index change, as shown in Equation (2), and the shapes of the spectrum including central wavelength and maximum intensity changed visibly as phase difference increased. When the recognition sites of the anti-CEA catch the target CEA, the catching process can induce the refractive index change of the reflected surface. Consequently, the phase difference between the horizontal and vertical polarizations varies with the refractive index change of the sample. In this way, the capture process can be monitored in real time through the central wavelength shifts of output spectrum.

A series of diluted glucose solutions experiments were carried out to determine the resolution of the weak measurement-based system and angular spectrum surface plasmon resonance (SPR), respectively. The angular spectrum SPR permits label-free detection and has high sensitivity. In this experiment, with the magnetron sputtering method, the prism was successively plated, 2 nm Cr and 50 nm Au, and obtained an SPR film layer structure. The resonance angle was 55° for water detection.

**Figure 4:**
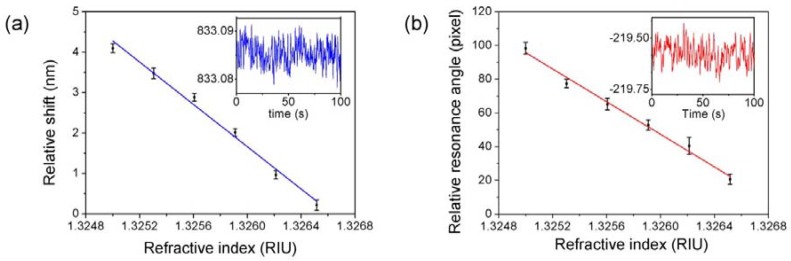
Experimental result for diluted glucose resolutions, (**a**) The result of the weak measurement-based system, inset: shifts measuring a 10 g/L glucose solution in 100 s; (**b**) The result of the angular spectra SPR based system. The error bar was obtained by repeating the measurements three times, inset: shifts measuring a 10 g/L glucose solution in 100 s.

In the weak measurement-based system, first, the wave plate was tilted to set the work point in the sharp area shown in Figure 2. Subsequently, a series of diluted glucose solutions with concentrations from 0 to 10 g/L were injected into the flow channel on the surface of the prism. As for the glucose solution, the relationship between the refractive index and concentration can be expressed as *n* = 1.325 + 1.515 × 10^−4^ C [28], where C is the concentration of glucose in grams per liter. We extracted the central wavelength shift in each concentration and compared it with the initial value in deionized water shown in Figure 4a. A 10 g/L glucose solution caused a shift of 3.88 nm in contrast to deionized water. The sensitivity characterized by δλ/δn is 2561 nm/RIU. After the system reached equilibrium, standard deviations of wavelength shifts were calculated by averaging the data of 100 s for different concentrations. The inset of Figure 4a shows shifts measuring a 10 g/L glucose solution; the standard deviation $\sigma_{s}$ is 0.002325 nm. Thus, the resolution defined by σ = $3\sigma_{s}/\delta \lambda /\delta n$ is calculated to be 2.7 × 10^−6^ RIU.

Within the same operations, Figure 4b displayed the results of the SPR-based system. A 10% glucose solution causes a shift of 77.3 pixel in contrast to deionized water. The sensitivity characterized by δλ/δn is 51,029 pixel/RIU. After the system reached equilibrium, standard deviations of resonance angle were calculated by averaging the data of 100 s for different concentration. The inset of Figure 4b shows the shifts measuring 10 g/L glucose solution, the standard deviation $\sigma_{s}$ is 0.05024 pixel. Thus, the resolution defined by *σ* = $3\sigma_{s}/\delta \lambda /\delta n$ is calculated to be 2.94 × 10^−6^ RIU. By contrasting the resolution to that calculated using SPR, the resolution of the weak measurement-based system is in the same order of magnitude and competes well with SPR.

In this experiment, to investigate the dynamic process for the recognition of CEA, a homemade flow channel was utilized, which was contacted with a peristaltic pump. The solution of analytes was pumped into the flow channel with a velocity of 100 μL/min. The flow setting was designed to ensure that the refractive index of the solution remained at a certain value. This was the only change in refractive index to occur during the reaction of the analytes. The analyte solution flowed through the channel and was captured by the recognition sites on the substrate, increasing the refractive index. The reaction process can be characterized by the phase difference corresponding to the central wavelength shift of the output spectra in terms of Equation (5).

It is well-known that silicon dioxide can be appropriately surface-functionalized for biosensing. In this work, dopamine was used for surface modification because of its versatile adhesion ability [29]. When the dopamine was injected into a homemade flow channel by peristaltic pump, it self-polymerized on the reflective surface of the prism to form a thin surface-adherent film that can be used for surface modification with a wide range of organic and inorganic materials. The dopamine solution is prepared at a concentration of 2 mg/mL buffered to 10 mM of Tris in the condition of pH 8.5.

In order to prevent temperature error, all buffers and reagents utilized in the experiment were placed in a water bath for 1 h in a 25 °C water bath kettle, and the laboratory environment was controlled at 25 ± 0.5 °C. First, a continuous infusion of PBS buffer into the flow channel at 100 μL/min.

The angle of the quarter-wave plates can be tilted according to the curve of the dip angle and central wavelength shift in Figure 2, for a working point with the most sensitive area. Starting recording data until the system become stable.

**Figure 5:**
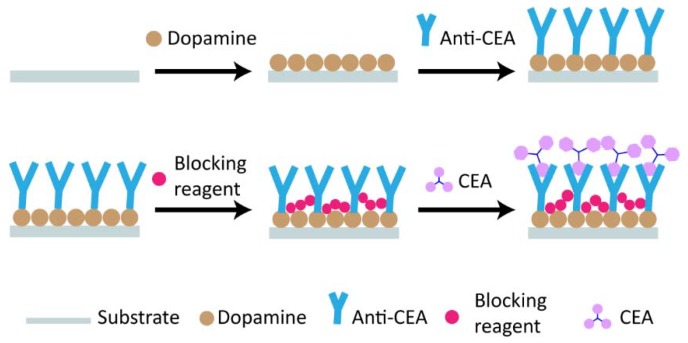
The schematic diagram of the detection process of CEA. Blocking reagent: protein-free blocking buffer, which can eliminate non-specific binding sites to avoid the non-specific adsorption.

The schematic diagram of the detection process of CEA is shown in Figure 5. A continuous infusion of fresh dopamine solution into the flow channel resulted in spontaneous deposition of a thin adherent polydopamine film on the surface of the substrate. Subsequently, PBS solution was injected into the flow channel to wash the remaining dopamine. Subsequently, the anti-CEA solution, which was prepared at a concentration of 50 μg/mL buffered to 10 mM of PBS at pH 7.4, was injected into the flow cell with a speed of 100 μL/min, and it could be adsorbed by the dopamine self-polymerization film. Subsequently, PBS solution was injected into the flow channel to wash the remaining anti-CEA. In order to avoid the non-specific adsorption, a protein-free blocking buffer, which could eliminate non-specific binding sites, was injected into the flow channel. Finally, CEA solution, which was prepared at a concentration of 50 μg/mL buffered to 10 mM of PBS in the condition with pH 7.4, was injected into the flow cell at a speed of 100 μL/min.

**Figure 6:**
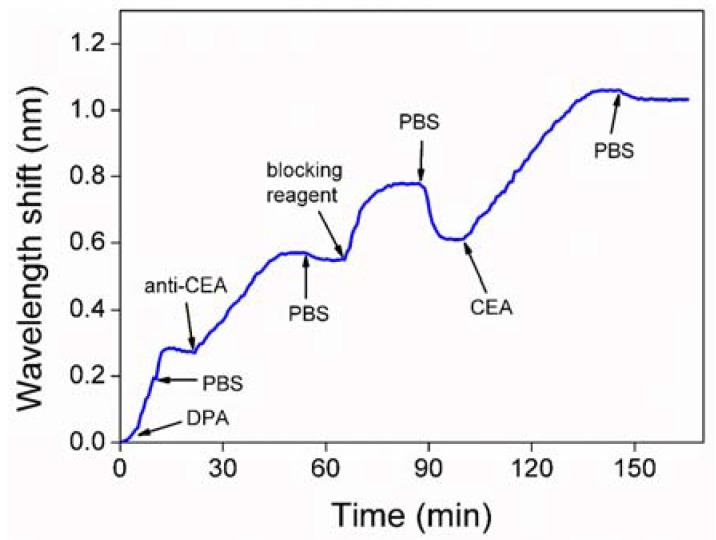
The result of detected process of CEA. DPA: dopamine solution; PBS: phosphate buffered saline solution; anti-CEA: CEA antibody; blocking reagent: protein-free blocking buffer.

The results of the detection process are shown in Figure 6; the *x*-axis represents the adsorption time and the *y*-axis denotes real-time shift in central wavelength relative to initial central wavelength. Dopamine self-polymerization formed a thin surface-adherent film that changed the refractive index of the substance touching the detection surface. The changed refractive index produced a phase difference when the test light passed through. Thus, as shown in Equation (1), the changed optical phase was detected through the central wavelength shift. After dopamine film deposition on the surface, a phosphate buffered saline (PBS, pH 7.4) solution causes a quick shift. The reason for such a quick shift is probably due to the body refractive index difference between DPA and PBS, since DPA buffered by Tris has no sodium chloride in it. Anti-CEA formed a layer of ligands on the dopamine film to capture CEA. Compared to the PBS solution, the protein-free blocking buffer caused a quick central wavelength shift. This quick response resulted from the body refractive index difference of the protein-free blocking buffer. Meanwhile, protein-free blocking buffer filled in the gap of the anti-CEA. The infusion of CEA antigen induced a central wavelength shift of 0.45 nm. Such a real-time kinetic curves of CEA detection is shown in Figure 6, which demonstrated that the recognition of CEA molecule could be monitored effectively with our weak measurement system.

This proposed protocol based on weak measurement is more simple and straightforward than other methods. It can be used to perform CEA detection without any need to label the test object. The interaction between tumor markers can be monitored in real time, which is significant for the quantitative analysis in cancer diagnosis. The feasibility of this weak measurement biosensor is demonstrated as described above. This method is greatly significant for the detection of cancer markers and pharmaceutical analysis.

**Figure 7:**
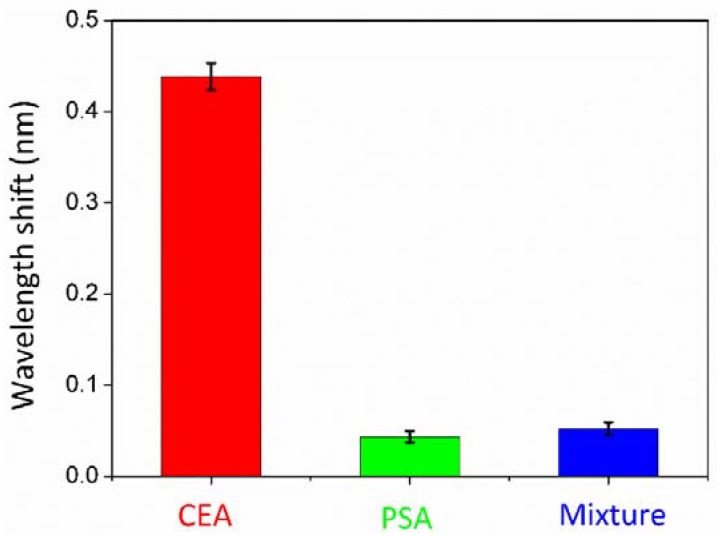
The central wavelength shifts of CEA and the two controlled trials. Error bars represent the standard deviations for three repeating experiments.

For the specificity verification, the experiment was designed and implemented as below. Dopamine was utilized for surface modification. After the infusion of anti-CEA, protein-free blocking buffer was injected into the flow channel to eliminate non-specific binding sites. Subsequently, the PSA solution was injected into the flow cell at a speed of 100 μL/min. The experiment for specificity demonstration was implemented with the same experimental setup as described above by replacing the CEA with PSA antigen. The CEA antigen was replaced with the mixture solution of CA125 and CA199 with the same concentration, with the same experimental setup as described above in Figure 7.

The results are shown in Figure 7. The central wavelength shift of the two controlled trials was far less than the central wavelength shift of CEA antigen detection experiment. It demonstrated that anti-CEA binding sites only specifically bound CEA, immune to PSA either the mixture of CA125 and CA199. Error bars represent the standard deviations for three repeating experiments.

To estimate the sensitivity of the sensor, the experiment was designed and implemented as below. The 50 μg/mL CEA antigen utilized as described above in Figure 4 was diluted to 25 μg/mL, 15 μg/mL, 10 μg/mL, and 5 μg/mL. The experiment setup implemented for Figure 4 was repeated with the CEA at concentrations of 25 μg/mL, 15 μg/mL, 10 μg/mL, and 5 μg/mL.

**Figure 8:**
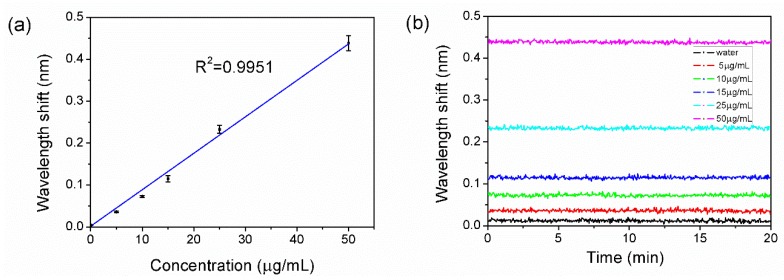
(**a**) The experimental result of CEA in different concentration: 50 μg/mL, 25 μg/mL, 15 μg/mL, 10 μg/mL, 5 μg/mL, and 0 μg/mL (water). Error bars represent the standard deviations for three repeating experiments. (**b**) The sensorgrams obtained with the different CEA concentrations reaction reaches equilibrium, and experimental data were collected over the course of 20 min.

The result is shown in Figure 8a, the central wavelength shift were proportional to the concentration of CEA, which exhibited the adsorption capacity of our sensor with different concentration. The sensorgram obtained with the different CEA concentrations reaction reached equilibrium, which is shown in Figure 8b. The stability of the system can be revealed by the standard deviation of experimental data collected over a period of time. The standard deviation $\sigma_{s}$ was calculate d to be 0.002146 nm with the data of 50 μg/mL in Figure 8b, showing a high robustness and stability. The limit of detection was determined as $3\sigma_{s}/\delta \lambda /\delta c$. δc is 50 μg/mL. δλ is 0.43 nm, which is the wavelength shift corresponding to the concentration of 50 μg/mL. $3\sigma_{s}/\delta \lambda /\delta c$ is calculated to be 0.749 μg/mL.

## 4. Conclusions

In this paper, we implemented the detection of tumor markers with a weak measurement system for the first time. The total internal reflection of the prism surface causes a phase difference between the two polarization components. In the weak measurement system, the central wavelength shifts of the output spectra are quantitatively correlated with the phase difference. Thus, the measuring parameter that induces the phase difference can be quantitatively analyzed by the central wavelength shift of the output spectra. The interaction between the target CEA and the recognition sites of the anti-CEA induces changes in the refractive indexes of the liquid sample, further leading to the varied phase difference between H polarization and V polarization. Therefore, the capture process can be detected in real time by monitoring the central wavelength shift of the output spectra. With the investigation of CEA, which was taken as an example of tumor markers to be detected, the feasibility and significance of this weak measurement sensing protocol were experimentally demonstrated. The experiments of PSA and mixture detection verified the specificity of the biosensor. In addition, the concentration detection of CEA was implemented for the sensitivity of the system. In conclusion, with the design of reflection-type configuration, the label-free detection of tumor markers can be monitored in real-time without gold film coatings on the detected area compared with SPR sensor. The weak measurement biosensor proposed in this work not only enriches the types of sensing methods for CEA detection, but also provides a potentially powerful solution for CEA-based molecular interaction, and the CEA-based molecular interaction will provide useful information for drug screening and drug analysis.

The limit of detection will significantly improve with higher power light sources and higher resolution spectrometers, in the future, if a line light source is used, multi-channel detection based on weak measurement can be easily implemented. This will be the focus of our group’s next project.
